# Universal Suppuration of the Cerebro-spinal Membranes, without Any Corresponding Symptoms

**Published:** 1843-08

**Authors:** 

**Affiliations:** Vienna


*Universal Suppuration of the Cerebro-spinal Membranes, without any corresponding symptoms.* By Professor Wagner, of Vienna.—Jacob Eichinger, a soldier in the 4th Light Infantry Regiment, had enjoyed good health during the seven years that he had been in the service. On the 17th of November, 1839, he was suddenly attacked with symptoms of gastric derangement, which increased on the following day, and compelled him to go to bed, at about a quarter to twelve, A. M. He fell asleep, and awoke in an hour delirious. Convulsions soon supervened; the man became comatose, and, although the most active antiphlogistic treatment was had recourse to, he died on the following day, November 19th, at half-past four in the morning.

The body was examined on the 20th. The cranial bones were remarkably thin, and the right side of the skull somewhat prominent. The whole of the superior surface of the cerebral hemispheres was covered with a layer of yellow fluid pus, and appeared somewhat flattened; no trace of the arachnoid could be found at this part. The pia mater was highly congested, and infiltrated with pus in its prolongations between the convolutions; the substance of the hemispheres was very much softened, and contained numerous points of blood when cut through; the lateral ventricles empty, and their walls softened in the highest degree; the pineal gland was very much enlarged, and did not contain any calcareous matter. The inferior surface of the cerebrum, and the whole of the cerebellum, were covered with pus, and extremely soft; the arachnoid here also appeared to have been destroyed; the base of the cranium was bathed in pus; no fluid in the third or fourth ventricles; the pineal gland much injected. The inner surface of the trachea was of a light red color, but the bronchi was healthy. There were some adhesions between the pleuræ, and the substance of the lungs was much congested; a few tubercles in the upper part of the left lung. The heart was very large, soft, and loaded with fat, but not diseased. In the abdominal cavity nothing worthy of notice was found. The bladder contained about half a pint of turbid urine. The fibrous membrane of the spinal marrow was much injected, and the cellular membrane particularly so; its whole surface, and especially opposite the cauda equina, was bathed in the same kind of purulent matter as the brain; there was no trace of the serous membrane, and the substance of the spinal marrow itself was converted into a thin, pultacious matter.

*Remarks.*—This remarkable case is almost unique in the annals of medical science. Pathologists must decide whether the inflammation commenced in the arachnoid membrane, or extended to it from the softened nervous tissue, or whether both states were simultaneously produced by one and the same cause. But, however this may be, we cannot but be struck with surprise that such extensive softening of the cerebro-spinal nervous mass, and universal suppuration of its serous membrane, should have existed without the production of any symptoms to indicate such extensive disease. Particular inquiries were made in the regiment in which the man had served, and it was ascertained that during the seven previous years he had enjoyed excellent health, having continued to do his duty as a soldier without interruption. It was only two days before his decease that gastric and convulsive symptoms made their appearance, and quickly terminated in coma and death.—*Prov. Med. Journ.*, Dec. 3. 1842.—*Ostr. Med. Woch.*, Nov. 4, 1842, from *Amer. Journ.*

